# A Nanomechanical Investigation of Three Putative Anti-Erosion Agents: Remineralisation and Protection against Demineralisation

**DOI:** 10.1155/2012/768126

**Published:** 2012-08-07

**Authors:** Ahmed Z. Abdullah, Anthony J. Ireland, Jonathan R. Sandy, Michele E. Barbour

**Affiliations:** ^1^Oral Nanoscience, School of Oral & Dental Sciences, University of Bristol, Bristol BS1 2LY, UK; ^2^Child Dental Health, School of Oral & Dental Sciences, University of Bristol, Bristol BS1 2LY, UK

## Abstract

An increasing interest in dental erosion as a clinical and scientific phenomenon has led to concerted efforts to identify agents which might protect against erosion. In this study, nanoindentation was used to investigate inhibition of erosive enamel demineralisation over time scales with direct clinical relevance. Nanohardness of polished human enamel specimens (*n* = 8 per group) was measured at baseline (B), after demineralisation (D1: citric acid, 0.3% w/v, pH3.20, 20s), after treatment (T), and after a second demineralisation (D2: as above). Data were analysed using repeated measures ANOVA. All specimens exhibited a similar reduction in nanohardness B-D1 in the range 35.2–39.5%. The positive control solution (saturated hydroxyapatite solution) and 4500 mg/L fluoride as NaF significantly increased nanohardness D1-T by 19.9% and 24.1%, respectively, whereas 1400 mg/L fluoride as NaF, casein phosphopeptide-amorphous calcium phosphate mousse and negative control (deionised water) had no significant effect. Nanohardness at D2 was indistinguishable for all groups, with total reduction in nanohardness B-D2 of 31.6% (4500 mg/L fluoride), 35.2% (positive control), 39.9% (1400 mg/L fluoride), 42.4% (negative control), and 43.7% (CPP-ACP product). In summary, 4500 mg/L fluoride significantly increased the nanohardness of previously demineralised enamel and resulted in the smallest total reduction in nanohardness but there were few statistically significant differences among the groups.

## 1. Introduction

Dental erosion is widely acknowledged as a common problem among children, adolescents, and adults [1]. There is a great deal of interest in the development and evaluation of treatments which might reduce the severity of erosion. Of these, salts of fluoride have received perhaps the most attention, and there is convincing evidence that applications of fluoride, particularly at high concentrations, can provide protection against erosion [2]. A number of different fluoride salts have been investigated, including stannous (tin) fluoride [3, 4], amine fluoride [3], titanium fluoride [5, 6], zirconium, and hafnium fluoride [6], but sodium fluoride has been the subject of the most studies owing to its widespread use in commercially available oral care products. 

Another agent which has received attention with regard to its ability to protect against erosion is a peptide of the bovine milk protein casein, casein phosphopeptide, in association with nanoparticles of calcium phosphate [7]. This is most commonly described in the literature and in marketing material as CPP-ACP (casein phosphopeptide-amorphous calcium phosphate). It has been incorporated, under the name Recaldent, into various products, one of the most successfully marketed being GC Tooth Mousse (Europe and Australia) or MI Paste (Japan, North and South America) (GC Corporation, Japan) [8]. 

Some investigations of agents which protect against erosion published in the research literature utilise prolonged exposures to the treatment agents. There are instances in which it could be argued that these are difficult to justify—it is hard to imagine the clinical relevance of a study where the tooth specimens are treated with a solution or paste for hours or even days before the erosive challenge is applied. Other studies utilise a cycling approach where the treatment and/or erosion/wear and/or saliva exposure are alternated with the intention of mimicking the changing oral environment over a period of several hours or days. Thus although in some cases the total exposure time to the treatment agent may be rather long, this is interspersed with acid and saliva exposures and thereby represents an accelerated aging model of erosion. In other cases the treatment may only be applied once and then a number of cycles of demineralisation and remineralisation are applied, in part to give sufficient erosive tooth loss to be detectable using laboratory techniques [9, 10]. As long as each exposure is of clinically relevant duration [11], these studies are much more justifiable and, while they may overestimate the amount of wear that would be seen clinically [12], are very useful in assessing the relative merits of protective strategies. There is also benefit in developing experimental models in which the treatment and erosion phases are very short, comparable to those seen in a single *in vivo* event, such as a single brushing of the teeth and/or a single intake of drink. Some recent examples of such studies can be found in references [13–15]. 

The aim of this study was to investigate three potentially erosion-preventive measures in a model which allows for single, clinically relevant exposure times. The experiment was designed such that we could assess both the effect of the treatments on the nanohardness of softened enamel (whether the treatment had caused any remineralisation) and whether the treatments affected the nanohardness of the same enamel after a subsequent, second erosive challenge (whether the treatment inhibited further demineralisation). 

## 2. Material and Methods

### 2.1. Specimen Preparation

Forty-four human enamel specimens were prepared by sectioning healthy, sound enamel from the mesial, distal, buccal, and lingual surfaces of 22 human premolars and molars, which had previously been sterilised by immersion in 20000 ppm available chlorine for 24 h, followed by immersion in 4% formaldehyde for 7 days, and subsequently long-term storage in 70% ethanol. The specimens were randomly assigned to five groups of 8 specimens. Specimens measuring 1-2 mm wide (around the perimeter of the tooth) and 2-3 mm long (along the central axis of the tooth) were sectioned from the teeth using a water-cooled, diamond tipped annular saw (Microsilice 2; Metals Research, Royston, UK) and mounted in epoxy resin (Stycast; Hitek Electronic Materials, Scunthorpe, UK) using silicone molds. The natural surface of the enamel was lapped using silicon carbide paper of 120 and 1200 grit size under slow water flow for the minimum possible time needed to remove any thin layer of resin that had flowed over the enamel surface and to remove the very outer enamel to provide a flat working surface. The specimens were then ultrasonicated in industrial methylated spirit (IMS) for approximately 2 min at room temperature to remove polishing debris and polished using an aqueous slurry of 0.25 *μ*m aluminium oxide to achieve a mirror finish and ultrasonicated again in IMS. All specimens were carefully inspected for lesions or damage before they were accepted for use in the study. Specimens were also assessed at D1 (see below) for their nanohardness and any outlying specimens, with nanohardness more than 1.5 standard deviations different from the mean, were eliminated and replaced with new specimens. This was done in order to exclude specimens that proved to be unusually susceptible, or resistant, to demineralisation and applied to 4 specimens (10%) in this study giving a final *n* = 40.

### 2.2. Nanoindentation

A nanoindentation system comprising a diamond-tipped Berkovich tip and vertical engagement with continual monitoring and control of vertical displacement was used (Triboscope; Hysitron Inc., Minneapolis, MN, USA) on an atomic force microscope (Nanoscope IIIa; Digital Instruments, Santa Barbara, CA, USA). Five widely spaced nanoindentations were performed on each specimen at each time point of the experiment and the mean from each specimen was used for statistical analysis. The AFM was used, with the Berkovich tip, to scan an area of 5 × 5 *μ*m prior to each indent to establish that the specimen was free from debris or microscopic scratches or cracks, and after indentation to check that the indent was equilateral and thus that the tip was engaged normal to the specimen surface. The nanoindentation data were analysed using Hysitron software using the Oliver & Pharr method to calculate nanohardness [16].

### 2.3. Study Regime

Specimens were treated as follows:

 Baseline nanohardness (B) measured on polished, untreated specimens
 D1: 1st demineralisation phase
 D1 nanohardness measured
 T: treatment phase with one of three test treatments or positive or negative control
 T nanohardness measured
 D2: 2nd demineralisation phase
 D2 nanohardness measured.

### 2.4. Demineralisation

The demineralising solution was 0.3% w/v citric acid monohydrate (Fisher Scientific, Loughborough, UK) adjusted to pH 3.20 using KOH. The specimens were attached to a disc 30 mm in diameter which was mounted on a quantitative overhead stirrer (R50D; CAT, Staufen, Germany) in order to provide a standardised method for agitation [17]. The angular velocity of the stirrer was adjusted to give an equivalent linear velocity of the specimens in the solution of 0.25 m/s. Specimens were exposed to acid for 20 seconds at room temperature. After each acid exposure, specimens were rinsed by immersion in deionised water for 60 seconds.

### 2.5. Treatment

Three test treatments were investigated alongside positive and negative control solutions.

#### 2.5.1. Test Treatments

Two sodium fluoride solutions, with fluoride concentrations of 1400 (pH 6.68) and 4500 (pH 6.74) mg/L (Fisher Scientific, Loughborough, UK) were used, and a CPP-ACP-containing mousse product, hereafter referred to as CPP-ACP product (GC Tooth Mousse, GC Corporation, Tokyo, Japan). 

#### 2.5.2. Control Solutions

The negative control was deionised water. The positive control was a solution saturated with respect to hydroxyapatite (HA), which was prepared by incrementally adding 0.1 g aliquots of HA powder (Sigma Aldrich, Gillingham, UK) to 1 L deionised water at 70°C under moderate stirring until no further hydroxyapatite dissolved. The solution was maintained at 70°C for 72 hours then left to cool slowly to room temperature and used immediately.

### 2.6. Exposure to Treatment Solutions

Specimens were attached to a disc to facilitate their handling. In the groups tested with fluoride or deionised water, specimens were immersed in the solution for 2 minutes without stirring. For the positive control, specimens were immersed without agitation for 60 minutes. The final group was covered with CPP-ACP product for 5 minutes according to the manufacturer's recommendation. After treatment, all specimens were rinsed with deionised water for 60 seconds.

### 2.7. Scanning Electron Microscopy

Scanning electron micrographs of enamel specimens at D2 were obtained using a Phenom scanning electron microscope (FEI, Netherlands) at nominal magnifications of 10000x and 20000x. 

### 2.8. Statistical Analysis

Nanohardness data were analysed by repeated measures ANOVA using SPSS statistical software package for Windows, version 16.0 (IBM Corporation, New York, USA).

## 3. Results

The mean nanohardness of the specimens as a function of stage and treatment and standard deviations are given in Table 1. In the reporting and discussion of the results, data are expressed as percent change from baseline nanohardness in order to provide a simple comparison of the effects of the treatments by eliminating the need for the reader to continually refer back to the baseline nanohardness. However, raw data can be seen in Table 1 to allow a comparison of the actual nanohardness values.

All specimens displayed a statistically significant softening from B to D1, with reduction in nanohardness in the range 35.2–39.5%. All specimens showed a numerical increase in hardness from D1 to T, but this was only statistically significant for the positive control (19.9% increase in nanohardness) and the 4500 mg/L fluoride (24.1% increase in nanohardness). All specimens showed a numerical softening from T to D2, but this was only statistically significant for 1400 mg/L fluoride (22.2% decrease in nanohardness) and CPP-ACP mousse (24.8% decrease in nanohardness). 

The overall reduction in hardness from B to D2 was CPP-ACP product (43.7%), negative control (42.4%), 1400 mg/L fluoride (39.9%), positive control (35.2%), and 4500 mg/L fluoride (31.6%). The statistical analysis indicated that there was a statistically significant difference between the positive and negative controls, but not between either control and the test solutions. 

**Figure 1:**
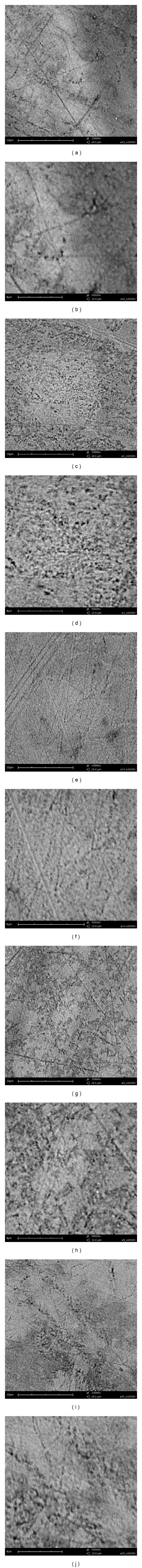
Scanning electron micrographs of enamel specimens after D2. (a) and (b) negative control; (c) and (d) 1400 mg/L F; (e) and (f) 4500 mg/L F; (g) and (h) CPP-ACP product; (j) positive control. Scale bars represent 10 *μ*m ((a), (c), (e), (g), (i)) and 4 *μ*m ((b), (d), (f), (h), (j)).

Scanning electron micrographs of a representative selection of the enamel samples are shown in Figures 1(a)–1(j). Areas of the specimens show the characteristic honeycomb pattern of etched enamel, interspersed with areas where the original polishing lines are apparent. This demonstrates that the etching was at an early stage without bulk tissue loss. No surface deposits were observed in any specimen group and all specimens from all treatment groups had a similar appearance. 

## 4. Discussion

In this *in vitro* study we investigated three agents with respect to their capacity to protect human enamel against dietary acid-mediated demineralisation. The use of nanoindentation allowed us to employ exposure times for acid and treatment that are relevant to a single clinical application, as was recommended in a recent thorough review of laboratory erosion and abrasion models [12]. The study design allowed the investigation of whether the test agents increased the nanohardness of previously demineralised enamel, and also whether the test agents protected the enamel against subsequent demineralisation.

Nanohardness was used as the outcome measurement and is interpreted as an indication of the extent of demineralisation that had taken place. Nanoindentation is recognised as one of the most sensitive methods for investigating enamel demineralisation [10, 14]. It has been shown that enamel nanohardness reduces as a function of acid exposure time and thus can be used as an indication of the extent of erosionlike demineralisation that has taken place up to exposure times of around 5 minutes [14, 18]. A correlation between nanohardness and the mineral content of calcium and phosphate-like species PO_x_H_y_ in demineralised human enamel has been previously demonstrated [19], and so nanoindentation was selected for use in this study. This is because it offers the possibility to investigate enamel demineralisation and remineralisation at very early stages, using time scales of relevance to clinical exposure [20], where the bulk tissue is still in place, and where only localised mineral depletion within the enamel structure has occurred, as indicated in Figure 1 [18]. 

Experimental conditions for the treatment stage of the study were, likewise, designed to some extent to mimic the clinical situation. The fluoride solutions were applied for 2 minutes, since the recommendation for toothbrushing is typically to brush for 2 minutes, and the concentration of fluoride in saliva is quite rapidly depleted after the brushing ceases. That is not to say each tooth receives 2 minutes' brushing—the figure is more likely to be of the order of 5 seconds [12]—but there will be an elevated concentration of fluoride in the mouth throughout the brushing process. The fluoride concentrations were chosen as representative of mass market toothpastes (1400 mg/L) and comparable to prescription-only products (4500 mg/L). The CPP-ACP product was applied according to the manufacturer's guidelines, by smoothing onto the enamel surface and allowing it to remain undisturbed for 5 minutes. The positive control, saturated hydroxyapatite solution, was applied to the enamel specimens for 60 minutes to represent a period of inactivity during the day where the tooth surface is bathed in saliva. 

The results indicate that 2 minutes' exposure to 4500 mg/L fluoride solution significantly increased the nanohardness of demineralised enamel, but 1400 mg/L fluoride solution did not. Furthermore, specimens treated with 4500 mg/L fluoride did not display significant softening when exposed to acid for a second time, whereas those treated with 1400 mg/L fluoride did soften significantly. Thus it appears that 4500 mg/L fluoride solution did, under these experimental conditions, provide both some rehardening of the enamel and some protection against subsequent demineralisation. We would interpret these results as signifying that the 4500 mg/L fluoride solution caused some precipitation of mineral within the softened surface enamel and that this resulted in both an increase in hardness and a reduced susceptibility to subsequent dissolution. This may suggest that the mineral deposited was fluorhydroxyapatite rather than calcium fluoride, and the moderately low concentration of fluoride and near-neutral pH of the treatment solution would support this inference [21]. The scanning electron micrographs in Figures 1(e) and 1(f) would also appear to offer support to this hypothesis, as they did not reveal any evidence of deposits *on* the surface, for instance of calcium fluoride. It should be noted, however, that the increase in enamel nanohardness afforded by treatment with 4500 mg/L fluoride was somewhat modest, and the resultant nanohardness after stage T was still some 18% lower than at baseline. The 1400 mg/L fluoride solution was either unable to produce such mineral deposits or those that were effected were insufficient to significantly increase nanohardness or reduce subsequent softening. A number of authors have sought to investigate the protective effects of fluoride compounds, and particularly sodium fluoride, against dental erosion [2]. A dose response effect showing increased protection at elevated sodium fluoride concentration has been observed both *in vitro *[13, 22–24] and *in situ *[25], although other studies have failed to reveal a dependence on fluoride concentration [26, 27].

Specimens that were demineralised and then treated with CPP-ACP product for 5 minutes, as recommended by the manufacturer, did not display a significant increase in nanohardness; neither did the treatment with CPP-ACP product provide any protection against subsequent demineralisation. There was no evidence of any of the product on the surface after D2 (Figures 1(g) and 1(h)). In a recent study, a significant increase in enamel microhardness was observed after treatment with the same CPP-ACP product [28], but the minimum exposure time investigated was 2 weeks. The contact time with the CPP-ACP product was, therefore, some 4000 times longer than in the present study. Another study using a 15-minute exposure to CPP-ACP demonstrated that enamel wear, as distinct from softening, was reduced by this treatment [29]. On the other hand, CPP-ACP product also failed to protect enamel against a subsequent erosive challenge in a model using 5 cycles of treatment, erosion, and remineralisation, although it was able to provide some protection when applied in conjunction with fluoride [30]. In a study where enamel specimens were eroded, placed in the mouth to facilitate remineralisation, treated with CPP-ACP product for 3 minutes applied either *ex vivo* or *in situ*, and finally replaced in the mouth for a further period of remineralisation, the CPP-ACP product did not confer any significant rehardening or protection of the enamel specimens [31]. It is plausible that putative protective effects of CPP-ACP are time dependent, with a significant effect only becoming apparent with long and/or repeated exposures. 

## 5. Conclusion

Only two treatments significantly rehardened the softened enamel (from D1 to T): the positive control and 4500 mg/L fluoride (as sodium fluoride). The CPP-ACP product, 1400 mg/L fluoride and negative control solutions did not reharden the enamel. The total reduction in nanohardness (from B to D2) was 31.6% (4500mg/L fluoride), 35.2% (positive control), 39.9% (1400 mg/L fluoride), 42.4% (negative control), and 43.7% (CPP-ACP product), but over the entire time course the repeated measures ANOVA indicated that only the positive and negative controls differed to a statistically significant degree.

## Figures and Tables

**Table 1:** Mean nanohardness and standard deviations (GPa) of human enamel specimens as a function of stage and treatment. B: at baseline, D1: after first demineralisation, T: after treatment, D2: after second demineralisation.

Treatment	Mean nanohardness (GPa) (Standard deviation)			
	B	D1	T	D2
Negative control	3.98 (0.37)	2.58 (0.32)	2.76 (0.35)	2.29 (0.25)
1400 mg/L F	4.25 (0.66)	2.82 (0.32)	3.28 (0.68)	2.55 (0.28)
4500 mg/L F	4.12 (0.52)	2.56 (0.27)	3.38 (0.54)	2.82 (0.49)
CPP-ACP product	4.30 (0.53)	2.78 (0.36)	3.22 (0.39)	2.42 (0.27)
Positive control	4.72 (0.46)	2.86 (0.29)	3.56 (0.50)	3.06 (0.42)
